# Case report: High-risk acute promyelocytic leukemia and COVID-19-related myocarditis one patient, two cytokine storms

**DOI:** 10.3389/fonc.2023.1095154

**Published:** 2023-04-11

**Authors:** Alexandra Ghiaur, Cristina Doran, Bogdan Ionescu, Lacramioara –Andreea Mohorea-Neata, Camelia Stancioaica, Roxana Hirjan, Aurelia Tatic, Mihaela Cirstea, Didona Vasilache, Dana Tabac, Ioana Lupescu, Daniel Coriu

**Affiliations:** ^1^ Department of Hematology and Bone Marrow Transplant, Fundeni Clinical Institute, Bucharest, Romania; ^2^ Division of Infectious Diseases, Fundeni Clinical Institute, Bucharest, Romania; ^3^ Carol Davila University of Medicine and Pharmacy, Bucharest, Romania; ^4^ Department of Radiology, Fundeni Clinical Institute, Bucharest, Romania

**Keywords:** acute promyelocytic leukemia, COVID-19, myocarditis, differentiation syndrome, immunomodulators

## Abstract

Acute promyelocytic leukemia (APL) is a unique, highly curable subtype of acute myeloid leukemia, owing to the therapeutic advances of the last decades which led to high complete remission rates and excellent long-term survival. Nevertheless, it remains associated with high early mortality rates. Early death is the major cause of treatment failure in APL and is mainly attributed to coagulopathy, differentiation syndrome, and less commonly, infectious events. Timely recognition of each complication plays a crucial role in the management of patients diagnosed with APL. Coronavirus Infectious Disease 2019 (COVID-19) has shown great heterogeneity in patient presentation. Clinical manifestations range from asymptomatic disease to severe forms, mainly characterized by a hyperinflammatory syndrome leading to acute respiratory distress and multiorgan failure. Patients with acute leukemia and concomitant COVID-19-related hyperinflammatory syndrome have particularly poor outcomes. We hereby report the case of a 28-year-old male patient who was diagnosed with high-risk APL, with severe associated coagulopathy at presentation. He was treated with chemotherapy according to the AIDA regimen. The first week of induction therapy was complicated by a differentiation syndrome manifesting as fever not attributable to infection and respiratory distress with pulmonary infiltrates, resolved after ATRA discontinuation and corticotherapy. On the fourth week of treatment, he tested positive for acute respiratory syndrome coronavirus 2 (SARS-CoV-2) with minor pulmonary involvement. Clinical manifestations over the following days included tachycardia and hypotension, associated with elevated inflammatory markers and cardiac biomarkers (troponin I x58 upper NV). Cardiovascular magnetic resonance imaging was consistent with myocarditis. COVID-19-associated myocarditis was successfully treated with methylprednisolone, intravenous immunoglobulins and Anakinra. Differentiation syndrome and COVID-19-associated myocarditis are two life-threatening complications that adversely impact survival. However, early recognition and prompt treatment initiation can improve clinical outcomes, as was the case of our patient.

## Introduction

Acute promyelocytic leukemia (APL) is a distinct type of acute myeloid leukemia, with a unique molecular pathogenesis as well as a characteristic clinical and biological presentation. The diagnostic hallmark of APL is the presence of a balanced translocation t(15;17)(q24.1;q21.1); rare cases of APL caused by variant translocations and gene rearrangements have also been reported.The oncogenic fusion protein PML-RARα acts as an aberrant retinoid receptor that blocks retinoic acid-induced myeloid differentiation (1) and represents a molecular target for differentiation agents such as the all-trans retinoic acid (ATRA). Additionally, the detection of the PML-RARα transcript in the bone marrow or peripheral blood through molecular analysis by reverse transcriptase polymerase chain reaction (RT-PCR) is a useful marker for monitoring the measurable residual disease (MRD) during consolidation therapy and after treatment has concluded.

Historically, the greatest progress in APL treatment is represented by the introduction of the ATRA and arsenic trioxide (ATO) combination, with or without associated chemotherapy. Nowadays, the choice of treatment regimen depends on disease risk stratification at diagnosis. Non-high-risk APL patients are defined as presenting with a white blood cell (WBC) count lower than 10×10^9^/L; in their case, the standard-of-care chemotherapy-free regimen of ATRA and ATO leads to complete remission rates of 90-100% (2, 3). However, for high-risk patients (presenting with WBC counts over 10×10^9^/L) the optimal approach is still a matter of debate, the two main approaches being ATRA+chemotherapy or the triple association of ATRA+ATO+chemotherapy (4) There are also trials suggesting that combination regimens associating ATRA and ATO with the anti-CD33 antibody-drug conjugate gemtuzumab ozogamicin could be a successful upfront approach in high-risk APL (3). In Romania, arsenic trioxide is not approved for the upfront treatment of high-risk APL, consequently the only choice of induction protocol consists of ATRA and chemotherapy.

APL is a medical emergency which requires immediate hospitalization and treatment initiation as soon as the diagnostic suspicion arises (5). Several studies have demonstrated that disease curability exceeds 80% in patients who survive induction (2, 3). However, early mortality estimates range from 5% in clinical trials to as high as 60% according to real-world data (6). The main causes of this high early mortality rate remain thrombo-hemorrhagic complications, infectious events and differentiation syndrome (DS).During the first month of induction therapy, APL patients therefore require careful monitoring aimed at identifying subtle changes in their clinical or biological state, in order to prevent major complications and early death.

Since March 2020, the novel severe acute respiratory syndrome coronavirus 2 (SARS-CoV-2) pandemic has brought unforeseen challenges to the worldwide healthcare system,significantly impacting both healthcare workers and their patients, as well as our society as a whole. SARS-CoV-2 is a RNA coronavirus that can affects various organ systems by binding to the angiotensin converting enzyme-2 (ACE2). While respiratory symptoms remain the clinical mainstay of the infection, other cells expressing high amounts of ACE2 include myocytes and the vascular endothelium, which explains in part the occurence of cardiovascular complications such as thrombosis, cardiomyopathy, myocarditis, arrhythmias, and acute myocardial injury in SARS-CoV-2 positive patients (7).

According to the existing literature, patients with acute myeloid leukemia who tested positive for SARS-CoV-2 had a higher mortality rate than those affected by other hematological malignancies (8). Risk factors for developing severe COVID-19 in the setting of a hematological cancer include neutropenia (PNN <0.5x10^9^/L), high C-reactive protein levels (> 20mg/dl), older age, disease status at the time of infection, and recent cytotoxic chemotherapy (8–10). Troponin elevation and a sepsis-induced coagulopathy (SIC) score ≥4 are poor prognosis indicators for hospitalized patients with COVID-19 (11–13).

## Case report

**Figure 1:**
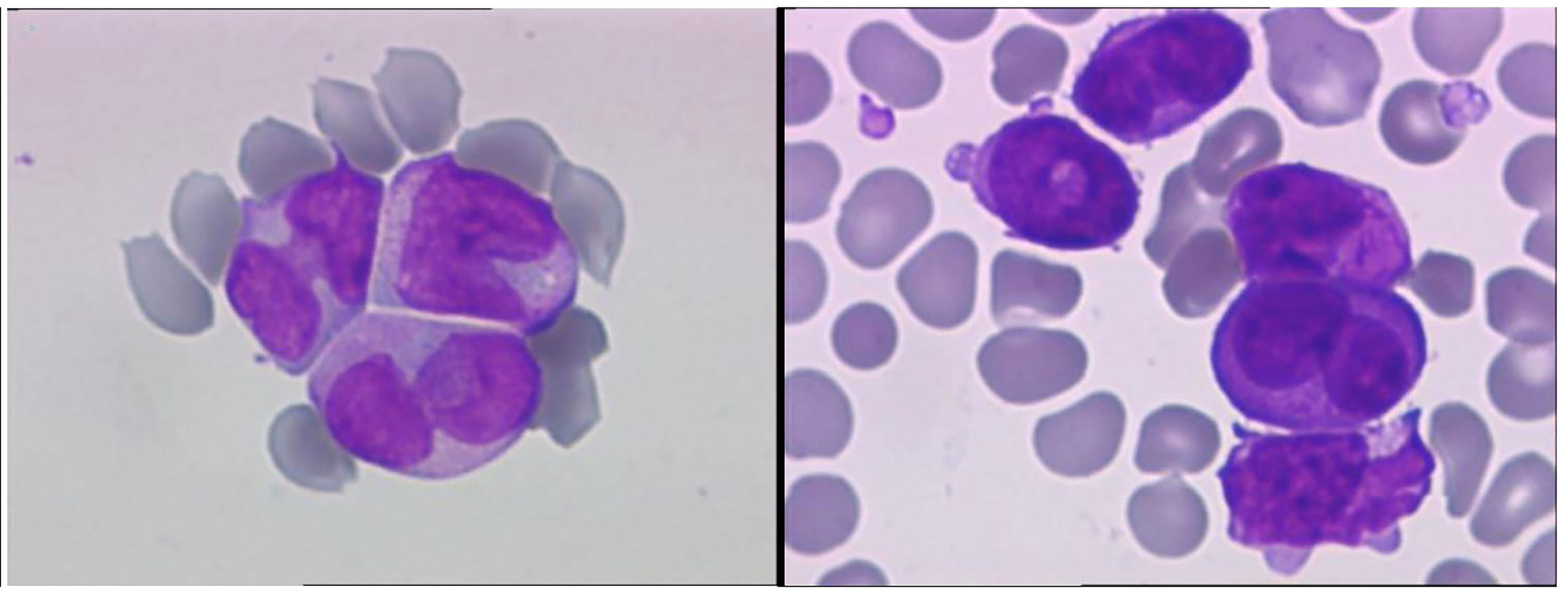
A typical promyelocytes with rare Auer rods, bilobed and reinform nucleus (MCG, x100).

A 28-year-old male with a history of smoking and no previously known medical conditions, vaccinated against SARS-CoV-2 six months before (BNT162b2 vaccine, 2 doses), presented with spontaneous bruising. He was referred to our hematology unit on October 1^st^, 2021. On admission, his total white blood cell count (WBC) was 137.53 x 10^9^/L with 92% atypical promyelocytes on the peripheral blood smear, hemoglobin 12.6 g/dL, and platelet count 45 x 10^9^/L (**Figure 1**, **Table 1**). Coagulation studies revealed decreased fibrinogen levels (115 mg/dL), an elevated INR (1.89) and D-dimers over 20,000 ng/ml (normal value<500 ng/ml). Flow cytometry assessment of a bone marrow sample showed 87% atypical promyelocytes. Fluorescence *in situ* hybridisation (FISH) identified the presence of a t(15;17)(q24;q21) translocation, while molecular analysis by RT-PCR was positive for the bcr2 isoform of the PML-RARα fusion protein.

**Table 1:** Laboratory parameters trends during hospitalization.

	Day l of induction chemotherapy	Day 5 differentiation syndrome	Day 21 SARS-COV2 positive	Day 27 Acute myocarditis	Day 48 Discharge	Normal value
WBC (xl09/L)	137.53	67	5.14	0.74	2.49	4.19-9.43
ANC (xi09/L)	3.97	6.7	2.19	0.44	1.32	2-7.15
Ly (xl09/L)	5.46	4.5	1.9	0.28	0.71	1.16-3.18
PLT (x109/L)	45	60	40	59	459	150-400
Fibrinogen (mg/dl)	115	90	260	618	500	200-400
0-dimers (ug/ml)	>20,000	>20,000	1,060	7,800	600	0-500
CRP (mg/L)	12	39	2,2	191	4	0-5
CK (U/L)	–	–	–	1,304	101	34-145
CK-MB {U/L)	–	–	28	17	14	0-24
Troponin I (ng/L)	–	–	–	19,765	196	0-34

WBC, white blood cell count; ANC, absolute neutrophil count; Ly, lymphocytes; PLT, platelet count; CRP, C-reactive protein.

After completion of the workup, the patient was diagnosed with high-risk Acute Promyelocytic Leukemia and disseminated intravascular coagulation. Induction treatment was started with ATRA 45 mg/m^2^/day divided in two daily doses and Idarubicin 12 mg/m^2^ on days 2, 4, 6, together with dexamethasone 2.5 mg/m^2^ BID for the prevention of differentiation syndrome, as well as blood product support (fresh-frozen plasma, cryoprecipitate, platelet transfusions, fibrinogen concentrate).

On day 5 of the induction protocol, he developed a fever and respiratory failure, with interstitial lung opacities on radiographic imaging and a SpO_2_ of 85% on RA. He had also gained around 3 kg in weight over the previous 48 hours. A transthoracic echocardiography found minimal pericardial effusion around the right atrium and a left ventricular (LV) ejection fraction of 60%. In the absence of positive bacterial cultures, an infection was considered unlikely and differentiation syndrome was diagnosed. ATRA was consequently withheld and the patient received treatment with dexamethasone 10mg BID, diuretics, and supplemental oxygen by nasal cannula, with quick remission of the symptoms and of the radiographic lung opacities.

**Figure 2:**
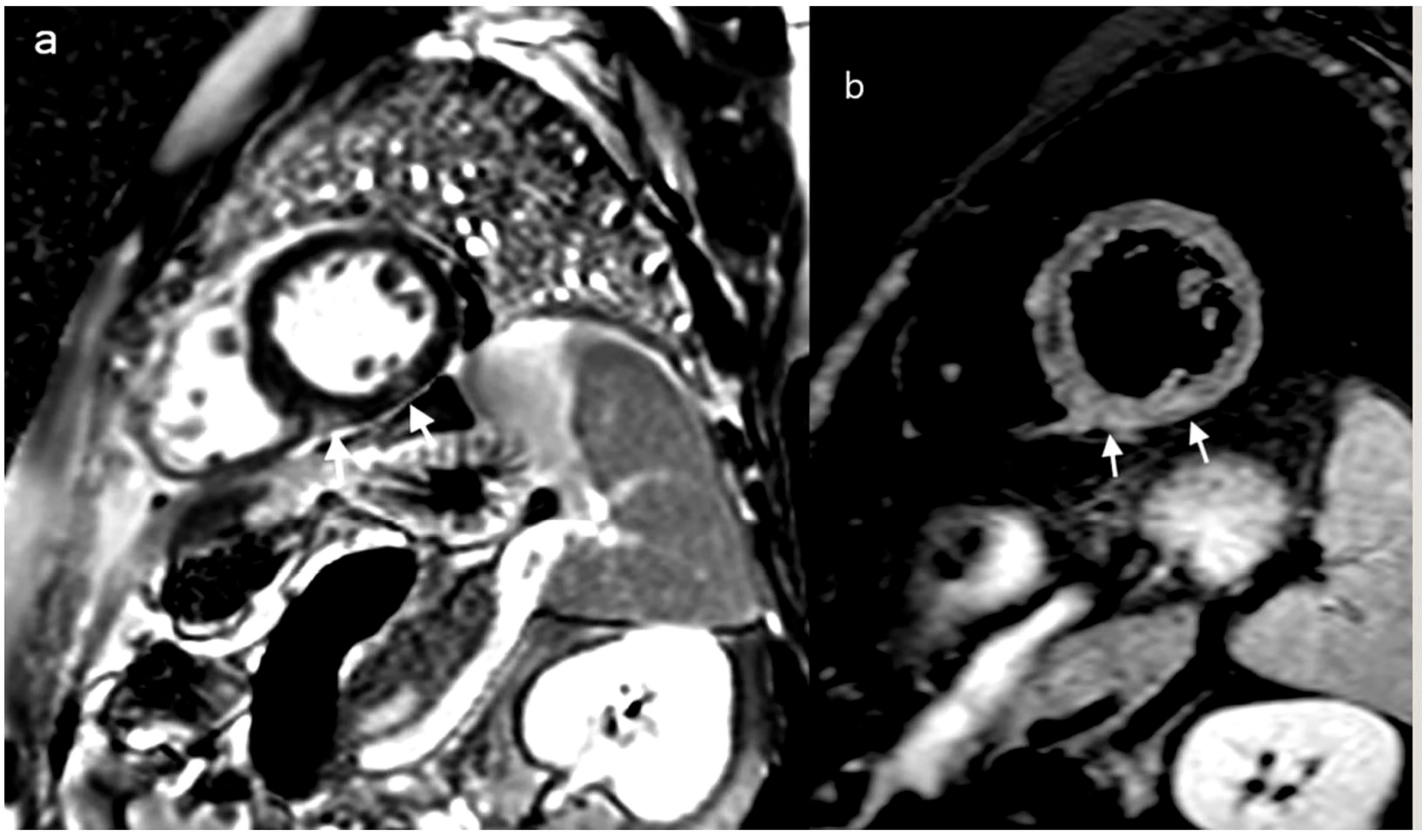
Cardiac MRI: delayed MRI enhancement sequence using the short-axis **(A)** highlights two contrast upateks with mid-myocardial and sub-epicardial topography at the level of the infero-septal and inferior wall of the left ventricle (white arrows) which correspond to focal ares of mycocardial edema visible in T2 weighted image with fats saturation **(B)** white arrows, suggesting acute myocarditis.

**Figure 3:**
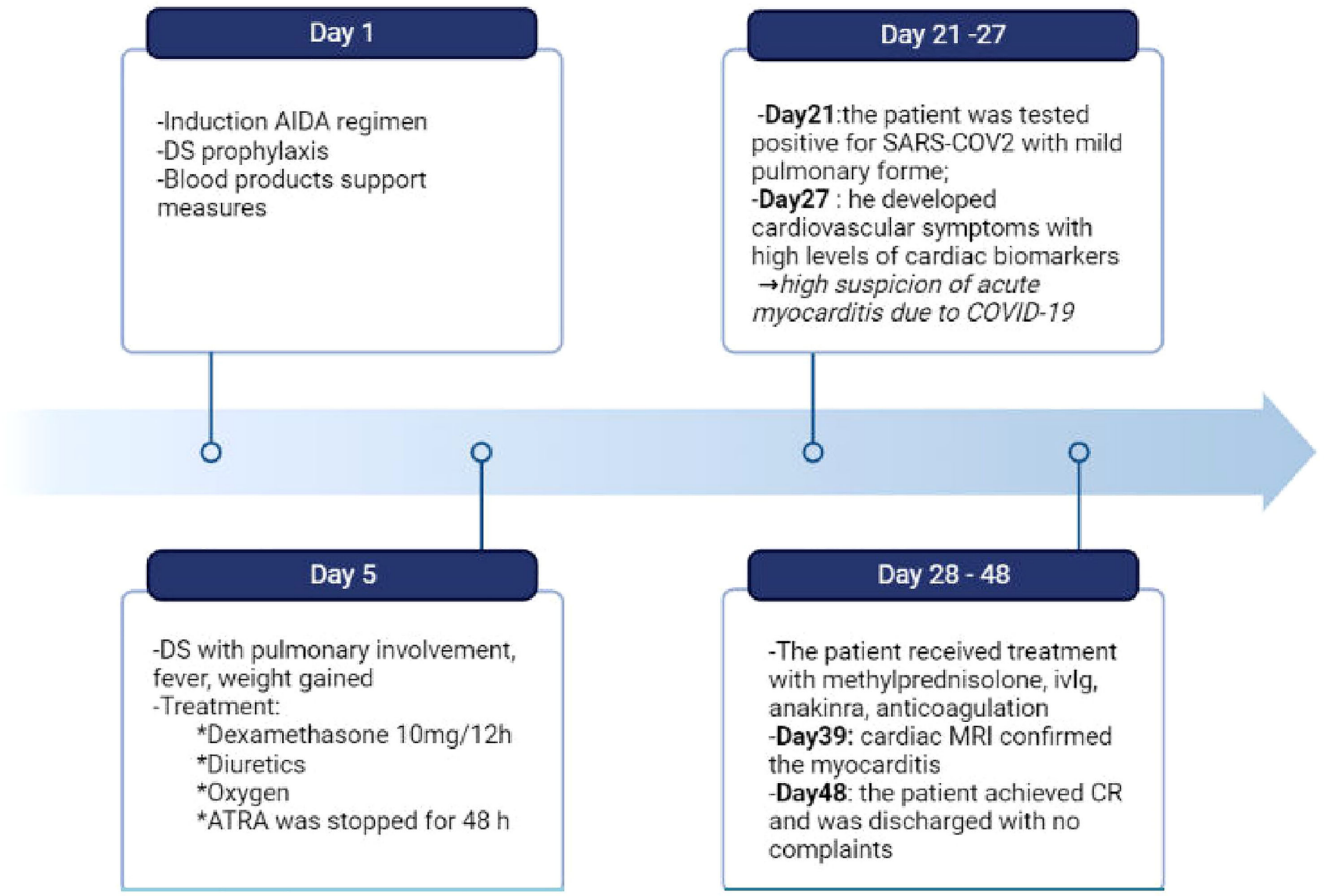
Timeline of clinical course of our patient from the day of admission to the day of discharge. During induction the patient experienced differentiation syndrome and covid-19 myocarditis, which required complex therapeutic approaches. DS, differentation syndrome; ivIg, intravenous immunoglobulin; CR, complete remission. Created with BioRender.com.

On day 21 of treatment, the patient developed a cough and subsequently tested positive for SARS-CoV-2 by RT-PCR, at which point Remdesivir was promptly started. Chest CT scans showed only minimal lung involvement consisting of a one-centimeter ground-glass area in the right lung base. In spite of this, his clinical status was in decline, with hypotension (Median Arterial Pressure = 59 mmHg), tachycardia, tachypnea, and persistent fever. On day 27 of induction (6 days after the positive SARS-CoV-2 test), laboratory studies revealed blood lactate levels of 3 mmol/L, BE= -6.5 and CRP levels reaching 191 mg/L (0-5 mg/l), with cardiac troponin I (TnI) levels up to 19,000 ng/L (0-34 ng/L) and increasing D-dimers (**Table 1**). Repeated ECGs showed mild ST depression in precordial leads V3-V6 and a sinus tachycardia ranging from 90 to 180 beats/min. No signs of pulmonary embolism were found on a chest angio-CT, also we rule-out an acute coronary syndrome. Given the high index of suspicion for myocarditis in the setting of very recent SARS-COV2 infection, the patient was administered methylprednisolone 125 g/day and intravenous immunoglobulins 15 g/day for three consecutive days. While COVID-19 cytokine storm was suspected, only minor elevations in serum IL-6 levels were found (7.7 pg/mL against a normal range of 0-5.9), with increased ferritinemia (8 times the normal upper limit); Anakinra was therefore preferred to Tocilizumab, although IL-1 levels could not be assessed in our institution. Additionally, given the context of neutropenic fever with clinical worsening, broad-spectrum antibiotherapy was administered throughout.The bacterial/fungal cultures came back negative. The patient’s clinical status steadily improved as his symptoms resolved over the following days. Myocarditis was later confirmed by cardiac magnetic resonance imaging performed 11 days after the onset of cardiac symptoms, which found focal edema within the septum, with prolonged myocardial relaxation times in T1-weighted mapping (1098 ms, normal range 885-1059) and T2 mapping (73 ms, normal range 45-65),a left ventricular ejection fraction(LVEF)of 50%, extracellular volume (ECV) 40% (**Figure 2**). On day 48 since the start of induction treatment our patient was discharged, having achieved morphologic complete remission and no longer presenting any cardiac or infections symptoms (**Figure 3**, **Table 1**).

## Discussion

### APL and DS

APL-DS is a cytokine-mediated systemic inflammatory response syndrome (14). It can develop at any point from 0 to 46 days following ATRA introduction, with a median of 12 days (15). Two decades ago, Frankel et al. described the first case series of 9 patients diagnosed with ATRA syndrome (16). Its pathophysiology is still incompletely understood and its clinical manifestations are highly polymorphic; they include unexplained fever, pulmonary infiltrates and respiratory distress, renal failure, rapid weight gain, cardiac failure, hypotension, pleural and pericardial effusions (17). The pathogenesis of DS is complex and is characterised by the release of pro-inflammatory cytokines (interleukin-1β (IL-1β), IL-6, IL-8, and tumor necrosis factor(TNF)-alpha) by differentiating blasts cell, resulting in a systemic inflammatory response syndrome (SIRS), capillary leak syndrome, and endothelial damage (18, 19). On the other hand, *in vitro* studies have shown that ATRA induces alterations in cell adhesion leading to the formation of promyelocyte aggregates, which may explain the occurence of microcirculation occlusions and leucostasis (20, 21).

According to existing studies, the incidence of DS varies widely between 2% to 48% (15), with an average DS-related mortality rate of 1% (4, 20). As DS is a life threatening complication and one of the main causes of early death in APL, rapid recognition of its signs and symptoms is of the utmost importance. Two forms of the syndrome have been described: early-onset DS and late-onset DS. In their 2013 paper, Montesino et al. highlighted that early-onset DS is associated more frequently with pulmonary infiltrates and weight gain, while hypotension, pericardial effusion, and renal failure are more common in patients with late-onset DS (19).

In the case of our patient, respiratory failure with an interstitial pulmonary infiltrate on chest radiograph, an episode of fever and weight gain occured at day 5 of induction treatment. APL-DS has no distinguishing clinical or laboratory features; therefore, our differential diagnosis included (without being limited to) sepsis, cardiac failure, and alveolar haemorrhage. We considered this a moderate form of early-onset DS and our patient was treated with dexamethasone 10mg BID, oxygenotherapy, and diuretics. Meanwhile, ATRA was discontinued for 48 hours, permitting the resolution of the respiratory symptoms and the disappearance of the aforementioned pulmonary infiltrate. For DS prevention we used prophylactic dexamethasone and the patient received idarubicine from the first day of addmision.

On day 21 of induction therapy the patient was diagnosed with a mild respiratory form of the SARS-CoV-2 infection, with minimal pulmonary involvement. However, six days later he developed cardiovascular symptoms – most likely related to the recent viral infection.

### APL and COVID-myocarditis

The pathogenesis of myocarditis in the setting of a SARS-CoV-2 infection is still incompletely understood, but is currently believed to be caused by a combination of direct viral injury and immune-related myocardial damage due to. t COVID-19-related cytokine storm. The clinical course of the infection has been divided into three separate stages: the upper respiratory tract phase (I), the pulmonary phase (II), and the ensuing hyperinflammatory state (III) (22). In our patient, cardiac abnormalities developed during the cytokine storm of hyperinflammatory state. Some of the risk factors that increase the likelihood of cardiac injury include smoking or recent exposure to anthracycline chemotherapy. In the setting of anthracycline administration, cardiotoxicity has been found to begin as early as the first dose of chemotherapy, although the compensatory mechanisms of the heart prevent it from becoming symptomatic until later stages (23). Very probably for our patient the recent anthracycline exposure could be a risk-factor for further development of cardiac complications. There are some controversies regarding the diagnosis and the management of COVID-19 myocarditis, particularly in a immunocompromised patient. In our case report, the diagnosis of COVID-19 myocarditis was based on the association between clinical presentation, the timing of onset of the events (cardiovascular symptoms following a COVID-19 diagnosis), laboratory tests (elevated troponine, CK, CK-MB, levels – **Table 1**) and imagistic findings on cardiac MRI (**Figure 2**). An endomyocardial biopsy was not performed.

Myocarditis is an inflammatory cardiac disorder without an ischemic cause. The most commonly identified causes of myocarditis encompass a wide range of human viruses, such as enteroviruses (coxsackievirus), adenovirus, parvovirus B19, herpesviruses, and most recently coronaviruses (including SARS-CoV-2). Other causes of myocarditis include bacterial(e.g. *Corynebacterium diphteriae, Borrelia burgdorferi*) and fungal infections, exposure to toxic compounds, chemotherapy (anthracyclins, immune checkpoint inhibitors), as well as systemic immune-mediated diseases (24).

In our case, we considered SARS-CO2 infection the main trigger for acute myocarditis. COVID-19 could play a direct role by myocardium infection (which can be proven by EMB) and an indirect role through a mecanism of citokine storm, immune dysregulation (cardiac symptoms occured on day 6 after initial diagnosis, during the hyperinflammatory phase) and immunothrombosis (coagulopathy of COVID19).

In the absence of a rapid therapeutic intervention, the initial acute myocardic inflammation may progress to tissue fibrosis, cardiac remodeling, and dilated cardiomyopathy (25). Studies have shown that the pro-inflammatory cytokine IL-1 plays a crucial role in the pathophysiologic mechanism of myocarditis (25). Additionally, several case reports support the use of the anti-IL1 monoclonal antibody anakinra for the treatment of myocarditis irrespective of etiology, describing a quick amelioration of cardiac function with a positive impact on patient outcomes (25–28). However, higher-level evidence examining the use of immunosuppressive treatment in the context of myocarditis is lacking.

Independently, methylprednisolone has been shown to have beneficial effects when started within 10 days of the onset of symptoms in patientswith biologically documented inflammation (CRP levels over 100mg/dl) (29).

Given the combination of a diagnosis of high-risk APL, the therapy-related immunosupression in a severely neutropenic patient, the marked elevations in inflammatory markers, and the recent administration of cardiotoxic chemotherapy, we decided to base its management on a combination of immunomodulatory treatments. Thus, our patient received concomittant corticotherapy, intravenous immunoglobulins and anakinra, leading to improvements in both his cardiovascular symptoms and cardiac enzyme levels. The patient was in a good condition with no cardiac-related clinical findings and he was followed by echocardiography initially monthly for the first 3 months and then every 3 months. Moreover, we perfomed an ECG twice per week during consolidation with ATO. His ECGs were within normal range, with no QTc prolongation or arrhythmia.

Given the cardiac complications which marked the course of the induction treatment and the possible long-term complications of COVID-19-related myocarditis, we continued the APL treatment following an ATRA-ATO-based regimen, foregoing anthracyclines during consolidation therapy.At the moment of writing, our patient has completed his treatment and has achieved MRD-negative remission. We have monitored the PML-RARA transcript after induction, after each cycle of consolidation and every 3 months post-consolidation. The patient achieved molecular remission after induction and continues to be undetectable for PML-RARA mutation.

### Coagulopathy

During hospitalization, our patient developed two distinct types of coagulation disorder. Firstly, he presented with a profoundly decompensated coagulopathy in the context of APL; this phenomenon is related to the expression of tissue factor and annexin A2 on the surface of abnormal promyelocytes causing both disseminated intravascular coagulation (DIC) and systemic hyperfibrinolysis. DIC management in the context of APL was based on platelet transfusions, fresh frozen plasma, cryoprecipitate and rapid introduction of ATRA, resulting in correction of the coagulopathy within 13 days.

Secondly, 6 days after having tested positive for COVID-19, our patient displayed a progressive worsening of his coagulation parameters, with D-dimers elevation up to 9100/uL (x 18 NV), prolonged activated partial thromboplastin time and INR (prothrombin time), and increased fibrinogen levels. No imagistic evidence of thrombosis was found. According to literature data, in-hospital mortality rates for patients with a SIC SCORE over 4, high D-dimer levels (>6 NV), high C-reactive protein levels, and elevated troponines are around 42% (11). In our case, we considered justified the introduction of anticoagulation in addition to the management of the underlying infectious cause.

Our patient had a complex clinical course with life-threathening complications such as APL-associated coagulopathy, differentiation syndrome, COVID-19 and acute myocarditis. The diagnosis of SARS-COV2 infection during induction of high-risk APL represents a particular challenge in terms of treatment and possible complications. There are few case reports published about COVID19 infection during induction of high-risk APL, none of them associated with acute myocarditis.

Our case report has several limitations. Firstly, genomic sequencing was not available in order to identify the precise SARS-COV2 variant responsible for infection. However, the patient become RT-PCR SARS-COV2 undetectable after 11 days and during October-December 2021 the dominant variant nationwide was Delta. Secondly, while bacterial or fungal cultures repeatedly came back negative, we have not tested for other viral coinfection or superinfection.

## Conclusion

We report a rare case of high-risk APL and COVID-19-related myocarditis occuring during induction therapy.

Since differentiation syndrome, cardiac complications, and infections occuring during APL induction treatment are the key causes of the high morbidity and mortality ratesin this highly curable hematological disease, their early recognition and prompt management are paramount.

## Data availability statement

The original contributions presented in the study are included in the article/supplementary material. Further inquiries can be directed to the corresponding author.

## Ethics statement

Written informed consent was obtained from the individual(s) for the publication of any potentially identifiable images or data included in this article.

## Author contributions

AG managed the case, reviewed the published literature, designed, and drafted the manuscript. CD collected and interpreted the data. L-AM-N, MC, CS, RH, IL, DT, DV were involved in clinical management; AT, BI and DC critically revised the manuscript. All authors contributed to the article and approved the submitted version.
